# The Relationship between Religion, Spirituality, Psychological Well-Being, Psychological Resilience, Life Satisfaction of Medical Students in the Gaziantep, Turkey

**DOI:** 10.1007/s10943-024-02027-2

**Published:** 2024-03-21

**Authors:** Hatice Tuba Akbayram, Hamit Sirri Keten

**Affiliations:** Gaziantep University Faculty of Medicine, Department of Family Medicine, Gaziantep, Turkey

**Keywords:** Religion, Spirituality-psychological well-being, Medical students-turkey

## Abstract

Religion and spirituality have been associated with better psychological health. The present study aims to investigate the relationship between the psychological well-being, psychological resilience, life satisfaction and religion/spirituality. An online cross-sectional study was conducted at the Gaziantep University School of Medicine, Turkey. The data were collected by using Personal Information Form, Individual Religion Inventory (IRI), Psychological Wellbeing Scale (PWBS), Brief Psychological Resilience Scale (BPRS), and Satisfaction with Life Scale (SWLS). A total of 399 students participated in the study, 84% of them stated that they were Muslim. The perception of good psychological health was significantly higher among Muslims (32.7%) than non-Muslims (14.3%) (*p* = 0.013). The PWBS, BPRS, SWLS scores were significantly higher in those who attached very importance to religious/spiritual practices compared to those who attached little importance. While PWBS scores and BPRS scores did not differ, the SWLS scores was significantly higher in Muslims compared with the non-Muslims. A positive correlation was found between the IRI scores and PWBS (*r* = 0.446 *p* < 0.001), BPRS (*r* = 0.252 *p* < 0.001), and SWLS scores (*r* = 0.450 *p* < 0.001) for Muslim participants. The study showed that giving importance to religious/spiritual practices is associated with better psychological health.

## Introduction

Medical training is considered one of the most academically and emotionally difficult educational programs (Quek et al., 2019). During their training, medical students experience stress induced by different factors, such as information-overload due to the medical curriculum, long study hours, lack of leisure or rest time, challenging exams, competition with peers, fear of academic failure, family expectations, changes in lifestyle, and financial difficulties (Fawzy & Hamed, 2017). Many studies conducted worldwide have reported that medical students have poorer psychosocial well-being and experience higher levels of depression, anxiety, and other psychological problems than other students (Castaldelli-Maia et al., 2019; Erschens et al., 2019; Pacheco et al., 2017; Pokhrel et al., 2020). Chronic stress and psychological problems experienced by medical students can negatively affect their professional development and academic performance. These problems may continue after graduating from medical school (Hathaisaard et al., 2022). Therefore, it is important to reveal the factors associated with medical students’ positive psychology such as psychological well-being, psychological resilience and life satisfaction. In this context, religion and spirituality (R/S) may be effective factors.

R/S are different terms that are often used interchangeably in the literature. Religion refers to the service or worship of God or a supernatural being (Paul Victor & Treschuk, 2020). Religiosity means believing in a religious doctrine and following and practicing the rules of a religion (Mohaghegh et al., 2022). Spirituality, on the other hand, encompasses a broader understanding of sacredness and entails an inherent sense of interconnectedness with life and a higher power that transcends the individual (Vieten & Lukoff, 2022).

Beyond their role in matters of faith and religious practice, R/S have also been associated with various aspects of psychological health. Being religious has been reported to reduce risky health behaviors, such as an unhealthy diet, alcohol, smoking, drug use, unsafe sex, suicide attempts, and violent behavior (Jones, 2004). Having religious beliefs is associated with an increased sense of meaning in life, higher hope and self-esteem, optimism, and life satisfaction (Dein, 2018). Spirituality helps individuals develop a sense of purpose in life and effectively managing stress (Kobbaz et al., 2021). The World Health Organization’s psychological first aid guide for field workers emphasizes the importance of spirituality and the practice of religious rituals in overcoming difficult periods of life (WHO, 2011).

Psychological well-being is a positive emotional state that increases functionality in people’s individual and social lives (Luna et al., 2020). Psychological well-being generally encompasses positive thoughts and feelings in individuals’ lives. Two different theoretical perspectives explain psychological well-being. The eudaimonic approach refers to well-being as one’s ability to determine meaningful life pursuits and one’s best performance. The hedonic approach defines well-being according to pleasure and happiness (Kubzansky et al., 2018). Psychological resilience, one of the elements of positive psychology, refers to the ability to cope with life’s challenges and stress (Han, 2021). Subjective well-being is defined as the extent to which individuals evaluate themselves in general. Life satisfaction is an important cognitive indicator of subjective well-being (Reyes et al., 2020). Psychological well-being and life satisfaction of individuals who are constantly exposed to stress may decrease over time (Labrague, 2021). However, people who are psychologically resilient and manage to cope with life’s challenges are more likely to be satisfied with their lives (Reyes et al., 2020). Therefore, it is necessary for medical students to maintain their psychological well-being, be psychologically resilient and not lose their life satisfaction in the face of long-term stress during their clinical education. In this context, religion and spirituality can be considered as an internal potential power that protects and supports students’ psychology.

Few studies in the literature have investigated the impact of R/S on medical students’ psychological well-being, psychological resilience and life satisfaction. To our knowledge, no Turkish studies have investigated the psychological impact of R/S on medical students. The aim of the current study is to investigate the relationships between R/S and psychological well-being, psychological resilience, and life satisfaction in a sample of medical students in Turkey. Our research hypothesis is that R/S is expected to explain higher psychological well-being, psychological resilience, and life satisfaction in medical students.

## Methods

### Study Group and Design

This cross-sectional study was conducted between May 15 and June 15, 2022, at the Gaziantep University School of Medicine. The Gaziantep University School of Medicine is one of the largest medical school in southern Turkey. For the study, all medical students (*n* = 1773) were invited to participate in an online survey (Google forms) through their class representatives. The inclusion criterion was being 18 years of age or older and answering the questionnaire completely, while the exclusion criterion was receiving psychological treatment (psychological medication or therapy).

### Data Collection

The questionnaire consisted of a personal information form, the Individual Religion Inventory (IRI), Psychological Wellbeing Scale (PWBS), Brief Psychological Resilience Scale (BPRS), and Satisfaction with Life Scale (SWLS).

### Personal Information Form

The first seven questions of the personal information form, consisting of 13 questions, aimed to reveal the students’ sociodemographic characteristics (age, gender, marital status, and family income status), presence of a chronic disease, the status of psychiatric medication use or therapy, and whether they had any cohabitants.

The students were asked to self-report their physical and psychological health status by answering the question “How would you evaluate your ….…. health in general?” The responses were classified as poor (very bad/somewhat bad, fair) and good (somewhat good/very good) and analyzed (Yun et al., 2022).

Smoking and alcohol use were inquired with the question “Do you smoke or consume alcohol?” Responses were coded as “yes” and “no.”

Participants were asked to specify their religious beliefs from a list of options including Muslim, Christian, Jewish, Atheist, Deist, does not want to specify, and other. None of the participating students identified as Christian or Jewish. In this study, those who stated that they were Muslim were defined as “Muslim” and all other students (Atheist-Deist-other and those who did not want to specify their belief) were combined in one category and defined as “non-Muslim”.

To evaluate the importance of religious or spiritual practices (RSP) in daily life, ‘In general, how important are RSP (such as prayer, worship, helping other) in your daily life?’ the question was asked. Participants responded to using a 5-point Likert scale from “not at all important (1)’ to ‘very important’ (5). Responses were coded as “1” and “2” little, “3” somewhat,“4 and 5” very.

### Individual Religion Inventory (IRI)

The inventory aims to measure the level of influence of religion in individuals’ personal lives, their level of religious knowledge, and the importance they attach to religion. The IRI consists of six items (Table 1). (e.g., “I often read information about my religion” and “It is important for me to personally devote a certain amount of time to religious thought and prayers”). It is a five-point Likert-type measuring tool (“Very untrue of me” = 1; “Untrue of me” = 2; “Somewhat true of me” = 3; “True of me” = 4; and “Very true of me” = 5). The lowest score obtainable from the inventory is 6 and the highest score is 30. The IRI was developed by Zagumny et al. (2012) and was translated into Turkish and analyzed for reliability and validity by Ayten (2013). Higher scores on the scale reflect higher levels of individual religiosity. The Cronbach’s alpha internal consistency coefficient of the scale was 0.851.

**Table 1:** Individual religion inventory

Items	
1	I often read information about my religion.
2	I’m trying to understand my religion more.
3	Religion is important to me because it provides answers to many questions about the meaning of life.
4	My religious beliefs affect my entire view on life.
5	Religion affects all the pursuits in my life.
6	Personally, it is important for me to devote a certain amount of time to religious thoughts and prayers.

### Psychological Well-Being Scale (PWBS)

This scale was developed to measure and investigate social and psychological well-being by Diener et al. (2010). The Turkish validity and reliability study for this scale was conducted by Telef (2013). The scale consists of 8 items, each of which defines important elements of human functioning, such as positive relationships and enjoying a meaningful and purposeful life. The items on the PWBS are scored on a 7-point Likert scale ranging from “strongly disagree” (1) to “strongly agree” (7). All items are rated as positive. The lowest possible score on the scale is 8, while the highest is 56, with higher scores indicating higher levels of psychological well-being. The Cronbach’s alpha internal consistency coefficient of the scale was 0.80.

### Brief Psychological Resilience Scale (BPRS)

The scale was developed by Smith et al. (2008). The adaptation of this scale into Turkish was carried out by Doğan (2015). The five-point Likert-type scale (ranging from “1” = does not describe me at all to “5” = describes me very well) consists of 6 items and has a one-dimensional structure. Items 2, 4, and 6 are reverse-coded. High scores indicates that the individuals have high levels of psychological resilience. The Cronbach’s alpha internal consistency coefficient of the scale was 0.81.

### Satisfaction with Life Scale (SWLS)

The scale was developed by Diener et al. (2010). Turkish validity and reliability study for the scale was conducted by Dağlı and Baysal (2016). The scale consists of five items (e.g., “I am satisfied with my life”). While the original is a seven-point Likert-type scale, the Turkish adaptation is a five-point Likert-type scale. The “Strongly disagree” option on the scale is scored at 1; “Somewhat disagree” at 2; “Somewhat agree” at 3; “Agree” at 4; and “Strongly agree” at 5. The lowest score that can be obtained from the scale is 5 and the highest is 25. High scores indicates high levels of life satisfaction. The Cronbach’s alpha internal consistency coefficient of the scale was 0.88.

### Ethical Considerations

Approval for this study was obtained from the Gaziantep University School of Medicine Clinical Research Ethics Committee (Decision no: 2022/154 date: 11.05.2022) and permission from Gaziantep University School of Medicine Dean’s Office for the current study was obtained. The ethical principles stated of the Declaration of Helsinki and good clinical practice standards were applied. Informed consent was electronically obtained from the participating students.

### Statistical Analysis

Data were analyzed using the Statistical Package for Social Sciences (SPSS) 22.0 (IBM Inc., Chicago, USA). Number and percentage values were used to evaluate descriptive statistics, and the chi-square test was performed to compare analytical data. IRI, PWBS, BPRS, and SWLS scores were calculated as median with the interquartile range (IQR) because the data were not normally distributed according to the Kolmogorov–Smirnov test. Numerical variables were compared using Kruskal–Wallis and Mann–Whitney U tests. The relationship between the findings obtained from the IRI, PWBS, BPRS, and SWLS was analyzed using Spearman’s rank correlation coefficient. Finally, multiple regression analysis was performed to examine the effects of IRI, BPRS, and SWLS on PWBS. *p* < 0.05 was considered statistically significant.

## Results

The questionnaire was completed by 456 students, of which 57 students receiving psychological treatment were excluded from the study. Thus, a total of 399 students were included in the study. The mean age of the participants was 21.96 ± 2.12 (min 18–max 39 years). Of these participants were 47.9% males and 52.1% females. Most of the them were unmarried (99%), were living with their families (39.1%), and their family income was moderate or high status. It was found that 11% of the participants had chronic diseases. The prevalence of cigarette users and alcohol users were 23.1% and 19.8% respectively. Of the participants, 84.2% (*n* = 336) were Muslim, 5.5% (*n* = 22) were atheist, and 3.3% (*n* = 13) were deist. No students identified themselves as Christian or Jewish, and 3.5% did not specify their beliefs. Among the participants, 56.4% defined their physical health and 29.8% defined their psychological health as good. Participants’ sociodemographic characteristics and physical and psychological health self-assessments are shown in Table 2.

**Table 2:** Socio-demographic and some personal characteristics of the students (*n* = 399)

		n	%
Gender	Female	208	52.1
	Male	191	47.9
Marital status	Married	4	1
	Single	395	99
Class	Preclinic (1st, 2nd, 3rd grade)	243	60.9
	Clinic (4th, 5th, 6th grade)	156	39.1
Family Income	Low	97	24.3
	Middle	253	63.4
	High	49	12.3
Chronic disease	Yes	44	11
	No	355	89
People living with	Family	156	39.1
	Friend/friends	134	33.6
	Alone	103	25.8
	Relative	6	1.5
Smoke	Yes	92	23.1
	No	307	76.9
Alcohol	Yes	79	19.8
	No	320	80.2
Self-rated physically health	Poor	174	43.6
	Good	225	56.4
Self-rated psychological health	Poor	280	70.2
	Good	119	29.8
Religionial Belief	Muslim	336	84.2
	Christian	0	0
	Jewish	0	0
	Atheist	22	5.5
	Deist	13	3.3
	Other	14	3.5
	Not reported	14	3.5

It was found that 20.5% Muslim participants were smokers and 11% were alcohol consumers and 36.5% of non-Muslim participants were smokers and 66.7% were alcohol consumers (*p* = 0.006, *p* < 0.001, respectively).

Among the participants, 55.7% Muslim and 60.3% non-Muslim students described their physical health as good (*p* = 0.791). Furthermore, 32.7% Muslims and 14.3% non-Muslims described their psychological health as good (*p* = 0.013).

In addition, 28.1% participants stated that they attach very importance to RSP (30.4% for males and 26% for females; *p* = 0.063). The perception of good physical and psychological health was significantly higher among those who attached high importance to RSP compared with those who attached little importance [(*p* = 0.036, *p* < 0.001, respectively) (Table 3)].

**Table 3:** Comparison of the perceived physical/psychological health status with the level of being-not being a Muslim and attaching importance to religious/spiritual practices

		Self-rated physically health			Self-rated psychological health		
		Poor n (%)	Good n (%)	*p*	Poor n (%)	Good n (%)	*p*
Muslims		149 (44.3)	187 (55.7)	0.493	109 (32.4)	110 (32.7)	0.013
Non-muslims		25 (39.7)	38 (60.3)		27 (42.9)	9 (14.3)	
RSP Importance*	Little	67^a^ (38.5)	71^a^ (31.6)	0.028**	115^a^ (41.1)	23^a^ (19.3)	< 0.001***
	Somewhat	70^a^ (40.2)	79^a,b^ (35.1)		115^a^ (41.1)	34^a^ (28.6)	
	Very	37^b^ (21.3)	75^b^ (33.3)		50^b^ (17.9)	62^b^ (52.1)	

* Importance of Religious/Spiritual Practices in daily life

** The difference between the groups is between those who attach little importance and those who attach very importance

*** The difference between the groups is between those who attach little - somewhat importance and those who attach very importance

Of the Muslim participants, 24.7% stated that they attached little importance, 42.3% some importance, and 33% very importance to RSP, whereas the rates were 87.3%, 11.1%, and 1.6%, respectively, among non-Muslim participants (*p* < 0.001).

The rates of smokers and alcohol consumers among those who attached very importance to RSP were significantly lower (12.5% and 3.6%, respectively) than those who attached little importance to such practices (34.8% and 38.4%, respectively) (*p* < 0.001).

The median (IQR) scores of the participants were 41 (11) for PWBS, 15 (5) for BPRS, and 14 (6) for SWLS. Those who attached more importance to RSP had significantly higher PWBS, BPRS, and SWLS scores than those who attached little importance (*p* < 0.001, *p* = 0.002, and *p* < 0.001, respectively). The relationship between the degree of importance attached to RSP and PWBS, BPRS, and SWLS scores is shown in Table 4.

**Table 4:** Comparison of PWBS, BPRS and SWLS scores with the level of emphasis on religious/ spiritual practices

Importance of Religious/Spiritual Practices in daily life	PWBS		BPRS		SWLS	
	Median	*p*	Median	*p*	Median	*p*
Little	38 (32–42)		15 (12–18)		12 (9–15)	
Somewhat	40 (35–45)	< 0.001	15 (13–17)	0.002	14 (12–17)	< 0.001
Very	45 (40–48)		17 (14–19)		16 (14–19)	

*PWBS* psychological well-being scale; *BPRS* brief psychological resilience scale; *SWLS* satisfaction with life scale

No statistically significant difference was found between Muslim and non-Muslim participants in terms of PWBS and BPRS scores (*p* = 0.658, *p* = 0.842, respectively). SWLS scores of Muslim participants were significantly higher than those of non-Muslim participants (*p* < 0.001). The comparison of Muslim and non-Muslims in terms of PWBS, BPRS, and SWLS scores is shown in Table 5.

**Table 5:** Comparison of PWBS, BPRS and SWLS scores with the level of emphasis on religious or spiritual practices

	Religionial_belief	Median	Percentile 25–75	IQR	*p*
PWBS	Muslims	40	35–46	11	0.658
	Non-muslims	41	37–44	7	
BPRS	Muslims	15	13–18	5	0.842
	Non-muslims	15	12–18	6	
SWLS	Muslims	15	11–18	7	0.000
	Non-muslims	13	10–15	5	

*PWBS* psychological well-being scale; *BPRS* brief psychological resilience scale; *SWLS* satisfaction with life scale, *IQR* Interquartile range

In this study, the IRI scores of only Muslim students were assessed because there were no participants who reported affiliation to an Abrahamic religion, such as Christianity or Judaism.

The median (IQR) IRI score of Muslim participants was 23 (7). A significant positive correlation was found between the IRI scores and PWBS (*r* = 0.446 *p* < 0.001), BPRS (*r* = 0.252 *p* < 0.001), and SWLS (*r* = 0.450 *p* < 0.001) scores of Muslim participants (Table 6). No significant correlation was found between the IRI scores of Muslims and their gender (median (IQR) in males: 23 (7) and in females: 23 (6), *p* = 0.482). In Model 1, IRI, BPRS and SWLS were included as independent variables related to PWBS. IRI score (β = 0.119), BPRS score (β = 0.242) and SWLS score (β = 0.534) had an effect on a high PWBS score (Table 7).

**Table 6:** Corelation between IRI, PWBS, BPRS and SWLS scores among Muslim students (*n* = 336)

Variables	Number of Items	Cronbach α	Median (IQR)	1	2	3	4
1. IRI	6	0.915	23 (7)	1			
2. PWBS	8	0.873	40 (12)	0.446^**^	1		
3. BPRS	6	0.855	15 (5)	0.252^**^	0.481^**^	1	
4. SWLS	5	0.851	15 (7)	0.450^**^	0.660^**^	0.410^**^	1

*IRI* individual religion inventory; *PWBS* psychological well-being scale; *BPRS* brief psychological resilience scale; *SWLS* satisfaction with life scale, IQR Interquartile range

^**^p < 0.01

**Table 7:** The effect of IRI, BPRS, SWLS on PWBS among Muslim students (*n* = 336)

Model 1	Unstandardized Coefficients		Standardized Coefficients		
Variable	B	SE	β	t	*p*
IRI	0.210	0.074	0.119	2.853	0.005
BPRS	0.542	0.093	0.242	5.847	< 0.001
SWLS	1.066	0.090	0.534	11.878	< 0.001
R^2^ = 0.530					

*PWBS* psychological well-being scale; *IRI* individual religion inventory, *BPRS* brief psychological resilience scale; *SWLS* satisfaction with life scale

*B* Unstandardized regression coefficient, *SE* Standard error, *β* Standardized regression coefficient, *R*^*2*^ coefficient of determination

## Discussion

In this study, we investigated how religion/spirituality might affect psychological well-being, resilience, and life satisfaction in a sample of medical students in Turkey. Turkey is a country governed by a secular democracy, and most of its citizens are Muslims. In our study, most participants (84.2%) were Muslim and no student reported affiliation with another Abrahamic religion (Christianity and Judaism). In this study, no significant difference was found between the psychological well-being and psychological resilience of Muslim and non-Muslim students. However, life satisfaction was significantly higher in Muslims. The psychological well-being and psychological resilience scores were significantly higher for those who attached very importance to RSP in daily life compared with those who attached little importance. Only one-third of Muslim students attached very importance to RSP. In Turkey, following Islamic rules is dependent on personal choice. The findings of our study indicate that a significant proportion of Muslim students do not pay very attention to RSP. As a remarkable result of this study, there was significant relationship between Muslims’ religiosity (IRI scores) and psychological well-being, psychological resilience and life satisfaction.

The impact of R/S on students’ psychological well-being and resilience has been investigated in different countries. Salem et al. reported that a strong relationship between religiosity and psychological well-being in a study conducted in Pakistan with medical and non-medical students. (Saleem & Saleem, 2017). A recent study conducted in China with 2,119 university students from 119 universities found that religiosity had a positive influence on individuals during the pandemic (Hu & Cheng, 2022). A study conducted among Muslim university students in Pakistan showed that participation in religious activities, clarity of belief, religious preferences, and prayer were associated with psychological well-being (Saleem et al., 2021).

In a study conducted in India, it is reported that Christian and Muslim medical students, there was a positive association of mental well-being with spirituality but not with religiosity (Gheevarghese et al., 2019). Chiang et al. (2021) reported that a positive relationship between spiritual health and resilience in a study conducted with nursing students in Taiwan. In a study in Brazil, students who stated that they were not religious were found to have a numerically higher rate of burnout, although not at a significant level (Dias et al., 2022). The findings of our study and existing literature suggest a positive impact of R/S on psychological well-being and psychological resilience among medical students.

A recent systematic review and meta-analysis reported that each dimension of R/S was positively associated with life satisfaction (Yaden et al., 2022). In a study conducted among medical students, religious coping was identified as an effective coping strategy compared with various others, and it was reported to be the most important determinant of satisfaction with life (Haider et al., 2022).

In a study conducted with university students in Turkey, a significant relationship was found between attitudes and behaviors toward Islam and happiness levels (Francis et al., 2017). In Trinidad, a study found no difference in terms of life satisfaction between the “religious” and “non-religious” groups. However, the same study reported a significant positive relationship between religiosity, religious well-being, and life satisfaction (Habib et al., 2018). In contrast, in our study, life satisfaction was significantly higher among Muslim participants than non-Muslims. Consistent with the literature, we found a positive relationship among attaching importance to RSP, religiosity, and life satisfaction.

### Strength’s and Limitations of the Study

One of the important strengths of this study is the first study in Turkey to investigate the relationship between religion and spirituality and psychological well-being, psychological resilience and life satisfaction in medical students. The individual religion inventory used in the study was an international scale and was not a scale applied only to Muslims.

However, there are some limitations of the study. The study was conducted in a single medical school; therefore, the findings cannot be generalized. Most of the participating students were Muslim, which may have a determinant effect on our findings. Another limitation is that the data were obtained through a self-reported online questionnaire, which may lead to the possibility of response bias.

## Conclusion

The findings of our study show that attaching importance to RSP is associated with perceived good physical and psychological health, psychological well-being, psychological resilience, and increased life satisfaction. The level of life satisfaction among Muslim participants was higher than that among non-Muslims. Moreover, we found that among Muslim participants, psychological well-being, psychological resilience, and satisfaction with life increased as religiosity increased. In view of these findings, RSP is found to play a highly significant role in developing and improving their psychological health for medical students.
